# Three novel variants in the arylsulfatase A (*ARSA*) gene in patients with metachromatic leukodystrophy (MLD)

**DOI:** 10.1186/s13104-019-4773-3

**Published:** 2019-11-06

**Authors:** D. Hettiarachchi, V. H. W. Dissanayake

**Affiliations:** Human Genetics Unit, Faculty of medicine, University of Colombo, 25, Kynsey Place, Colombo 08, Sri Lanka

**Keywords:** Metachromatic leukodystrophy, ARSA, Neurodegenerative disorders

## Abstract

**Objective:**

To describe the genetic variants in the *ARSA* gene in Sri Lankan patients with metachromatic leukodystrophy (MLD). As the variant profile of MLD in the Sri Lankan population is currently unknown.

**Results:**

Twenty patients from eighteen Sri Lankan families were screened for *ARSA* gene mutations. We found 13 different genetic variants of these three were novel. The three novel variants were p.Asp281Asn, p.Asp283Asn, p.Ala344Asp. Seven patients out of 20 were also positive for the pseudodeficiency (PD) allele c.1049A>G (p.Asn350Ser). This is the first report to describe the molecular genetic variants of Sri Lankan patients with MLD.

## Introduction

Metachromatic leukodystrophy (MLD; OMIM #250,100) is a rare neurodegenerative disorder resulting in a deficiency of arylsulfatase A activity (ARSA, EC 3.1.6.8). This autosomal recessive condition has an incidence of 1 in 40,000 live births [1]. ARSA is a lysosomal enzyme involved in cerebroside sulfate degradation. Hence its deficiency results in the accumulation of cerebroside-3-sulfate in the lysosomes of the tissues of the central and peripheral nervous system [2]. High levels of sulfide cause progressive demyelination and loss of white matter with clinical features of neurodegeneration. MLD is classified in three subtypes depending on the age of onset as; infantile (0–2 years); juvenile with subtypes of early (3–6 years) and late juvenile (7–16) and adult. The onset of the adult form is after sexual maturity, sometimes not until the fourth or fifth decade of life [3].

MLD is known to result from a homozygous or a compound heterozygous mutation in the arylsulfatase A gene on chromosome 22q13. It’s a small gene of about 3 kb with eight exons encoding a 507 amino-acid precursor (GenBank accession numbers, NM_000487.4 and NP_000478.2) [4, 5]. This gene is transcribed into one major and two minor mRNA species of 2.1, 3.7 and 4.8 kb respectively [6]. Thus far 186 mutations in the ARSA gene have been reported among them 181 variants are directly associated with MLD (http://www.hgmd.cf.ac.uk/ac/gene.php?gene=ARSA). However, the variant spectrum in Sri Lanka is not known. Additionally, there are neutral variants known as the ARSA pseudodeficiency (Pd) allele where there is a reduction of ARSA activity. Here we describe the genetic variants in the *ARSA* gene in Sri Lankan patients with metachromatic leukodystrophy (MLD) as it is currently unknown.

## Main text

### Methods

#### Patients

Twenty patients from eighteen families were screened for *ARSA* gene mutations. Patients were recruited from the Human Genetics Unit (HGU), faculty of medicine university of Colombo and Asiri Surgical Hospital center for genomics. Based on the expert neurological opinion, neuroimaging findings such as Magnetic resonance imaging (MRI) studies and clinical diagnosis of MLD.

#### Molecular genetic analysis

Following written informed consent, the patient’s genomic DNA was extracted from the patient’s whole blood samples collected in K3EDTA tubes. DNA extraction was carried out using the QIAamp DNA Blood Mini Kit; Qiagen, according to the manufacturer’s protocol. The full length of the ARSA gene including coding exons and corresponding intron/exon boundaries were amplified by seven forward and reverse primer sets [7].

Amplified PCR fragments were checked by agarose gel electrophoresis to ensure it meets the quality criteria required to produce an accurate result. Then PCR fragments were subjected to sanger sequencing using the BigDye^®^ Terminator v3.1 Cycle Sequencing Kit by Applied Biosystems^®^ 3130 Genetic Analyzer followed by ethanol purification. The resulted ABI files were analyzed using codon code aligner software aligning with a human ARSA reference sequence obtained from ENSEMBLE database. Selection of the pathogenic and likely pathogenic variants and removing the benign variants from available variants in the patients. This was carried out using the information available on prior publications, data from population frequency databases and clinical databases.

### Results

We studied twenty patients from 18 unrelated families, all the patients were of Sri Lankan origin and referred to us from all parts of the island. The median age at diagnosis was 19.9 years (range = 0.8–40). The clinical categories were as follows infantile 7; early juvenile 1; late juvenile 2 and adult 10. We found 13 different amino acid changes in these three were novel (p.Asp281Asn, p.Asp283Asn, p.Ala344Asp) (Table 1). Seven patients out of 20 were positive for the pseudodeficiency (PD) allele c.1049A>G and showed a corresponding p.Asn350Ser amino acid change and low ARSA enzyme activity.

**Table 1:** Genetic variants in the ARSA gene in Sri Lankan patients with MLD

Patient number	Age range at diagnosis (years)	Phenotype	Nucleotide substitution	Amino acid change	Zygosity	Pseudo deficiency allele
01	0–5	Infantile	c.342C>T c.1055A>G	p.Arg114X p.Asn350Ser	Compound heterozygote	Present
02	6–10	Late juvenile	c.342C>T c.601T>C	p.Arg114X p.Tyr201His	Compound heterozygote	No
03	0–5	Infantile	c.1055A>G c.1109G>A	p.Asn350Ser pArg370Gln	Compound heterozygote	Present
04	0–5	Infantile	c.841G>A c.1055A>G	p.Asn350Ser *p.Asp281Asn*	Compound heterozygote	Present
05	36–40	Adult	c.1055A>G c.1115G>A	p.Asn350Ser and p.Arg372Gln	Compound heterozygote	Present
06	40–45	Adult	c.1055A>G c.1115G>A	p.Asn350Ser and p.Arg372Gln	Compound heterozygote	Present
07	0–5	Infantile	c.251C>T c.847G>A	p.Pro84Lys *p.Asp283Asn*	Compound heterozygote	No
08	6–10	Early juvenile	c.1178C>G c.1425C>A	p.Thr393Ser *p.Ala344Asp*	Compound heterozygote	No
09	0–5	Infantile	c.1178C>G c.1425C>A	p.Thr393Ser p.Ala344Asp	Compound heterozygote	No
10	0–5	Infantile	c.938G>A	p.Arg313Gln	Homozygote	No
11	30–35	Adult	c.251C>T	p.Pro84Lys	Heterozygote	No
12	30–35	Adult	c.847G>A	p.Asp283Asn	heterozygote	No
13	0–5	Infantile	c.256C>T c.847G>A	p.Arg86Trpp.Asp283Asn	Compound heterozygote	No
14	36–40	Adult	c.251C>T	p.Pro84Lys	Heterozygote	No
15	6–10	Late juvenile	c.1178C>G	p.Thr393Ser	Heterozygote	No
16	30–35	Adult	c.1055A>G	p.Asn350Ser	Heterozygote	Present
17	36–40	Adult	c.1055A>G	p.Asn350Ser	Heterozygote	Present
18	30–35	Adult	c.346C>T c.1178C>G	p.Arg116Ter p.Thr393Ser	Heterozygote	No
19	40–45	Adult	c.346C>T	p.Arg116Ter	Heterozygote	No
20	30–35	Adult	c.346C>T c.1178C>G	p.Arg116Ter p.Thr393Ser	Heterozygote	No

### Discussion

In our study, we found three novel amino acid changes p.Asp281Asn, p.Asp283Asn, p.Ala344Asp in the ASA protein. All three novel variants in the Sri Lankan population were compound heterozygous variants and were A>G, G>A and C>A transitions respectively. Among the patients who were positive for the pseudodeficiency (PD) allele c.1049A>G, p.Asn350Ser, five of them were compound heterozygous for the mutation and only two were heterozygous. The high frequency of the common PD allele was observed in the Sri Lankan population in line with already published data in other populations [8, 9]. The second most frequent mutation found in our study was c.1178C>G, p.Thr393Ser this mutation was found in almost all the clinical subcategories of MLD from juvenile to adult form. Other common mutations were p.Pro84Lys, p.Asp283Asn and p.Arg116Ter with three unrelated patients to each variant. Among these p.Pro84Lys and p.Arg116Ter has been reported to be pathogenic in other populations. However p.Asp283Asn has not been reported elsewhere Asp281 is highly conserved among arylsulphatases and its crystal structure shows an essential catalytic site which provides a magnesium ion binding site within the protein, we predict the change from aspartate to asparagine from a negatively charged amino acid to a polar amino acid (containing an amide group) disrupts the catalytic binding site to magnesium ions by adversely affecting protein folding. Other amino acid changes observed in two unrelated individuals each were p.Arg114X, p.Arg372Gln and p.Ala344Asp. Mutation p.Arg114X leads to an early stop codon with a resultant truncated ARSA polypeptide that is 114 amino acids long. Truncated proteins are classified as pathogenic according to ACMG guidelines [10]. This study has shed light on the molecular genetic variants found in MLD that have not been previously reported and given the diversity of the population this knowledge will add further to the understanding and timely diagnosis of MLD.

## Limitations

- This study only concentrates on patients clinically diagnosed to have MLD who underwent genetic testing.
- It is only a descriptive study of the variant profile in Sri Lankan patients.
- The effect of these variants on individual patients and their future treatment options needs to be considered.
- Functional studies and family member screening need to be performed to ascertain the exact pathogenicity of the novel variants.

## Data Availability

Data is not available in a public domain it will be shared on request from interested parties.

## Abbreviations

MLD: metachromatic leukodystrophy
ARSA: arylsulfatase A gene
HGU: Human Genetics Unit
