# Correction to: Three novel variants in the arylsulfatase A (*ARSA*) gene in patients with metachromatic leukodystrophy (MLD)

**DOI:** 10.1186/s13104-020-4908-6

**Published:** 2020-01-22

**Authors:** D. Hettiarachchi, V. H. W. Dissanayake

**Affiliations:** Human Genetics Unit, Faculty of Medicine, University of Colombo, 25, Kynsey Place, Colombo 08, Sri Lanka

## Correction to: BMC Res Notes (2019) 12:726 10.1186/s13104-019-4773-3

After publication of the original article [1], the authors became aware of a typographical error in the original Table 1. Nucleotide substitution c.1425C>A corresponding to amino acid change p.(Ala344Asp) should be corrected as c.1031C>A.

**Table 1:** Genetic variants in the ARSA gene in Sri Lankan patients with MLD

Patient number	Age range at diagnosis (years)	Phenotype	Nucleotide substitution	Amino acid change	Zygosity	Pseudo deficiency allele
01	0–5	Infantile	c.342C>T c.1055A>G	p.Arg114X p.Asn350Ser	Compound heterozygote	Present
02	6–10	Late juvenile	c.342C>T c.601T>C	p.Arg114X p.Tyr201His	Compound heterozygote	No
03	0–5	Infantile	c.1055A>G c.1109G>A	p.Asn350Ser pArg370Gln	Compound heterozygote	Present
04	0–5	Infantile	c.841G>A c.1055A>G	p.Asn350Ser *p.Asp281Asn*	Compound heterozygote	Present
05	36–40	Adult	c.1055A>G c.1115G>A	p.Asn350Ser and p.Arg372Gln	Compound heterozygote	Present
06	40–45	Adult	c.1055A>G c.1115G>A	p.Asn350Ser and p.Arg372Gln	Compound heterozygote	Present
07	0–5	Infantile	c.251C>T c.847G>A	p.Pro84Lys *p.Asp283Asn*	Compound heterozygote	No
08	6–10	Early juvenile	c.1178C>G c.1031C>A	p.Thr393Ser *p.Ala344Asp*	Compound heterozygote	No
09	0–5	Infantile	c.1178C>G c.1031C>A	p.Thr393Ser p.Ala344Asp	Compound heterozygote	No
10	0–5	Infantile	c.938G>A	p.Arg313Gln	Homozygote	No
11	30–35	Adult	c.251C>T	p.Pro84Lys	Heterozygote	No
12	30–35	Adult	c.847G>A	p.Asp283Asn	heterozygote	No
13	0–5	Infantile	c.256C>T c.847G>A	p.Arg86Trpp.Asp283Asn	Compound heterozygote	No
14	36–40	Adult	c.251C>T	p.Pro84Lys	Heterozygote	No
15	6–10	Late juvenile	c.1178C>G	p.Thr393Ser	Heterozygote	No
16	30–35	Adult	c.1055A>G	p.Asn350Ser	Heterozygote	Present
17	36–40	Adult	c.1055A>G	p.Asn350Ser	Heterozygote	Present
18	30–35	Adult	c.346C>T c.1178C>G	p.Arg116Ter p.Thr393Ser	Heterozygote	No
19	40–45	Adult	c.346C>T	p.Arg116Ter	Heterozygote	No
20	30–35	Adult	c.346C>T c.1178C>G	p.Arg116Ter p.Thr393Ser	Heterozygote	No

The corrected Table 1 is shown in this erratum.
